# Longitudinal dynamics of serum neurofilament light chain and prognostic value for functional outcome after acute stroke: a prospective cohort study

**DOI:** 10.3389/fneur.2026.1932102

**Published:** 2026-10-05

**Authors:** Guangyong Chen, Yuxin Zhu, Feng Chen, Changhao Zhang, Jin Huang, Weijie Wu, Lei Chen, Lingling Lin, Yueping Chen, Anqi Lin, Qinqin Liu, Peipei Wang, Lidong Zhu, Xuerong Huang

**Affiliations:** 1 Department of Neurology, The Third Affiliated Hospital of Wenzhou Medical University, Wenzhou, Zhejiang, China; 2 The First School of Medicine, School of Information and Engineering, Wenzhou Medical University, Wenzhou, Zhejiang, China; 3 The Second School of Medicine, Wenzhou Medical University, Wenzhou, Zhejiang, China; 4 Clinical Laboratory, The Third Affiliated Hospital of Wenzhou Medical University, Wenzhou, Zhejiang, China; 5 Renji College, Wenzhou Medical University, Wenzhou, Zhejiang, China; 6 Department of Rehabilitation Medicine, The Third Affiliated Hospital of Wenzhou Medical University, Wenzhou, Zhejiang, China

**Keywords:** biomarker, functional outcome, longitudinal dynamics, serum neurofilament light chain (sNfL), stroke

## Abstract

**Background:**

Serum neurofilament light chain (sNfL) is a blood biomarker of neuroaxonal injury, but its early longitudinal dynamics and prognostic value in clinically heterogeneous acute stroke populations remain insufficiently defined. This study characterized early sNfL changes and examined their association with functional outcome after acute stroke.

**Methods:**

This prospective cohort study consecutively enrolled 104 patients with acute stroke and 86 healthy controls (HCs) between January 2023 and December 2024. Patients were grouped as intracerebral hemorrhage (ICH, *n* = 28), ischemic stroke treated with intravenous thrombolysis (IS+IVT, *n* = 33), and ischemic stroke without intravenous thrombolysis (IS−IVT, *n* = 43). sNfL was measured within 24 h after onset (D1) and on day 7 (D7). Functional outcome was assessed by modified Rankin Scale (mRS) at 3 and 6 months. Linear mixed-effects models assessed longitudinal sNfL trajectories. Multivariable logistic regression assessed prognostic associations, with the 3-month outcome as the primary endpoint and the 6-month outcome as the secondary endpoint. Restricted cubic spline (RCS) and interaction analyses were exploratory.

**Results:**

sNfL increased significantly from D1 to D7 (*p* < 0.001) and was higher in patients with stroke than in HCs at D1 (*p* < 0.001). In the linear mixed-effects model adjusted for age and admission National Institutes of Health Stroke Scale (NIHSS) score, longitudinal sNfL trajectories did not differ significantly among the clinical stroke groups (time × group interaction, *p* = 0.225). The predominantly mild-to-moderate severity profile may have limited the detection of trajectory differences. In multivariable logistic regression, each 10-pg/mL increase in D1 sNfL was associated with 5% higher odds of poor functional outcome at 3 months (OR = 1.05; 95% CI, 1.00–1.12; *p* = 0.047) and 7% higher odds at 6 months (OR = 1.07; 95% CI, 1.01–1.14; *p* = 0.025). D7 sNfL was not independently associated with poor functional outcome after adjustment. Exploratory RCS analyses found no statistical evidence of nonlinearity for D1 sNfL and suggested a nonlinear association between D7 sNfL and 3-month poor functional outcome.

**Conclusion:**

sNfL levels increased during the first week after acute stroke. Higher D1 sNfL was associated with poor functional outcome at 3 and 6 months, suggesting potential value for early prognostic assessment.

## 1 Introduction

Stroke is a leading cause of death and long-term disability and remains a major public health challenge worldwide (1, 2). Epidemiological data indicate that stroke carries the second highest mortality burden among noncommunicable diseases globally (2). Beyond acute mortality, stroke is the third largest contributor to disability-adjusted life years (DALYs), accounting for approximately 5.7% (5.1%–6.2%) of the total global disease burden (1). This combined burden of death and chronic disability has substantial socioeconomic consequences for health care systems worldwide.

Pathophysiologically, stroke comprises two major, biologically distinct entities. Ischemic stroke (IS) is caused primarily by cerebral arterial occlusion, whereas hemorrhagic stroke results from vascular rupture and bleeding into the brain parenchyma (3). In IS, thrombosis or embolism interrupts cerebral blood flow and initiates a cascade involving energy failure, excitotoxicity, and secondary injury processes that ultimately lead to neuronal necrosis in the affected region (4). Hemorrhagic stroke results from vessel rupture, blood extravasation, and hematoma formation, with subsequent tissue injury and cell death related to mass effect, impaired local perfusion, perihematomal edema, and blood component–related toxicity (5, 6). Although both major stroke types can involve neuroaxonal injury, their initiating mechanisms and subsequent pathological evolution differ substantially, resulting in marked heterogeneity in acute treatment strategies (7–11).

In clinical practice, repeated assessment of stroke severity is central to treatment decisions and outcome optimization. This assessment currently relies mainly on clinical scales, particularly the National Institutes of Health Stroke Scale (NIHSS), which quantifies the severity of acute neurological deficits (12). However, the NIHSS primarily reflects clinical symptomatology rather than the biological process of neuroaxonal injury, and therefore cannot readily capture the ongoing evolution of axonal damage after stroke. This temporal gap between early clinical assessment and subsequent molecular events highlights the need for dynamic biomarkers that can monitor progressive neurological injury over time.

Neurofilament light chain (NfL) is a cytoskeletal protein with a relative molecular mass of approximately 68–70 kDa and is a major component of neuronal intermediate filaments. It is expressed predominantly in neurons and is enriched in large-caliber myelinated axons. NfL is widely used as a sensitive biomarker of neuroaxonal injury and degeneration (13). After neuronal injury, NfL is released into the extracellular space and can subsequently enter the cerebrospinal fluid and peripheral circulation; circulating concentrations may partly reflect the severity of axonal injury (13). Advances in ultrasensitive assay technologies have enabled reliable quantification of blood NfL, and its value for diagnosis, stratification, and prognostic assessment has been investigated across multiple neurological diseases (14–17). Because blood sampling is minimally invasive, repeatable, and suitable for longitudinal monitoring, NfL has potential utility for assessing disease activity and, in selected contexts such as multiple sclerosis, tracking treatment response; in stroke, longitudinal studies support its use mainly for monitoring injury dynamics and prognosis (7, 18, 19).

In stroke research, previous studies have suggested that blood NfL levels are associated with stroke severity and functional outcome, with many primary studies focusing on IS or intracerebral hemorrhage (ICH) separately (20–22). Systematic evidence further indicates substantial heterogeneity in stroke populations and sampling windows (7). Prospective cohorts that include both ischemic and hemorrhagic stroke and compare the prognostic value of NfL at different time points remain limited. It also remains insufficiently clarified whether longitudinal NfL trajectories differ independently among stroke types. This question is pathophysiologically relevant because ischemic and hemorrhagic stroke differ substantially in the mechanisms and subsequent evolution of axonal injury (8). In addition, previous studies have mainly evaluated NfL–outcome associations using conventional regression-based approaches (22–24), leaving the potential nonlinear dose–response relationship between NfL and functional outcome, as well as possible effect modification by stroke type or intravenous thrombolysis (IVT) status, insufficiently examined. These gaps may limit the interpretation of NfL in acute stroke, because its dynamic pattern is relevant to the selection of optimal sampling time points, interpretation of test results, and accuracy of risk stratification.

We therefore conducted a prospective cohort study to: (1) characterize early dynamic changes in serum NfL (sNfL) during the first week after acute stroke and compare trajectories across clinically relevant groups defined by stroke type and IVT status; (2) evaluate and compare the prognostic associations of sNfL on day 1 after onset (D1) and day 7 after onset (D7) with poor functional outcome at 3 and 6 months; and (3) examine the dose–response relationship between sNfL and poor functional outcome and assess whether this association differed across these groups.

## 2 Methods

### 2.1 Study design

This single-center prospective cohort study was conducted at the Third Affiliated Hospital of Wenzhou Medical University between January 2023 and December 2024. The hospital ethics committee approved the study protocol (approval No. YJ2022105). The study was conducted in accordance with the principles of the Declaration of Helsinki. Written informed consent was obtained from all participants or their legally authorized representatives before enrollment.

### 2.2 Study participants

Consecutive patients hospitalized in the neurology ward with clinically diagnosed acute stroke supported by neuroimaging were enrolled. Healthy controls (HCs) recruited during the same period served as the control group.

The inclusion criteria for patients with stroke were as follows: (1) age ≥18 years; (2) acute stroke diagnosed by neurologists based on acute focal neurological symptoms and supported by computed tomography (CT) or magnetic resonance imaging (MRI) findings consistent with cerebral infarction (10) or nontraumatic intracerebral hemorrhage (11); (3) admission within 24 h after symptom onset, with peripheral blood collected for sNfL measurement; and (4) complete NIHSS records at admission.

The exclusion criteria for patients with stroke were any of the following: (1) a history of stroke within the previous 6 months, neurodegenerative disease such as Alzheimer disease, Parkinson disease, or multiple sclerosis, or peripheral neuropathy; (2) a history of head or spinal cord trauma or surgery within the previous 3 months; (3) prestroke modified Rankin Scale (mRS) score > 2; or (4) incomplete sample collection, missing clinical data, or loss to follow-up.

HCs were screened according to the neurological disease-related exclusion criteria described above. For HCs, venous blood was collected once at enrollment, and no follow-up assessments were performed.

### 2.3 Screening and grouping of participants

During the study period, 129 patients with stroke were consecutively screened. Of these, 19 were excluded because D7 serum samples could not be obtained due to early discharge, transfer to another department, or refusal of further blood sampling. D1 sNfL was not measured in these patients, and the scheduled outcome assessments were not performed. The remaining 110 patients entered follow-up, of whom 6 were lost to follow-up. The final analysis included 104 patients. According to stroke type and IVT status, patients were categorized into three clinical stroke groups: ICH (*n* = 28), IS with IVT (IS+IVT, *n* = 33), and IS without IVT (IS−IVT, *n* = 43). These clinical stroke groups were used for all subsequent group-based trajectory and interaction analyses. During the same period, 86 HCs were recruited from community health screening programs as the control cohort (Figure 1).

**Figure 1:**
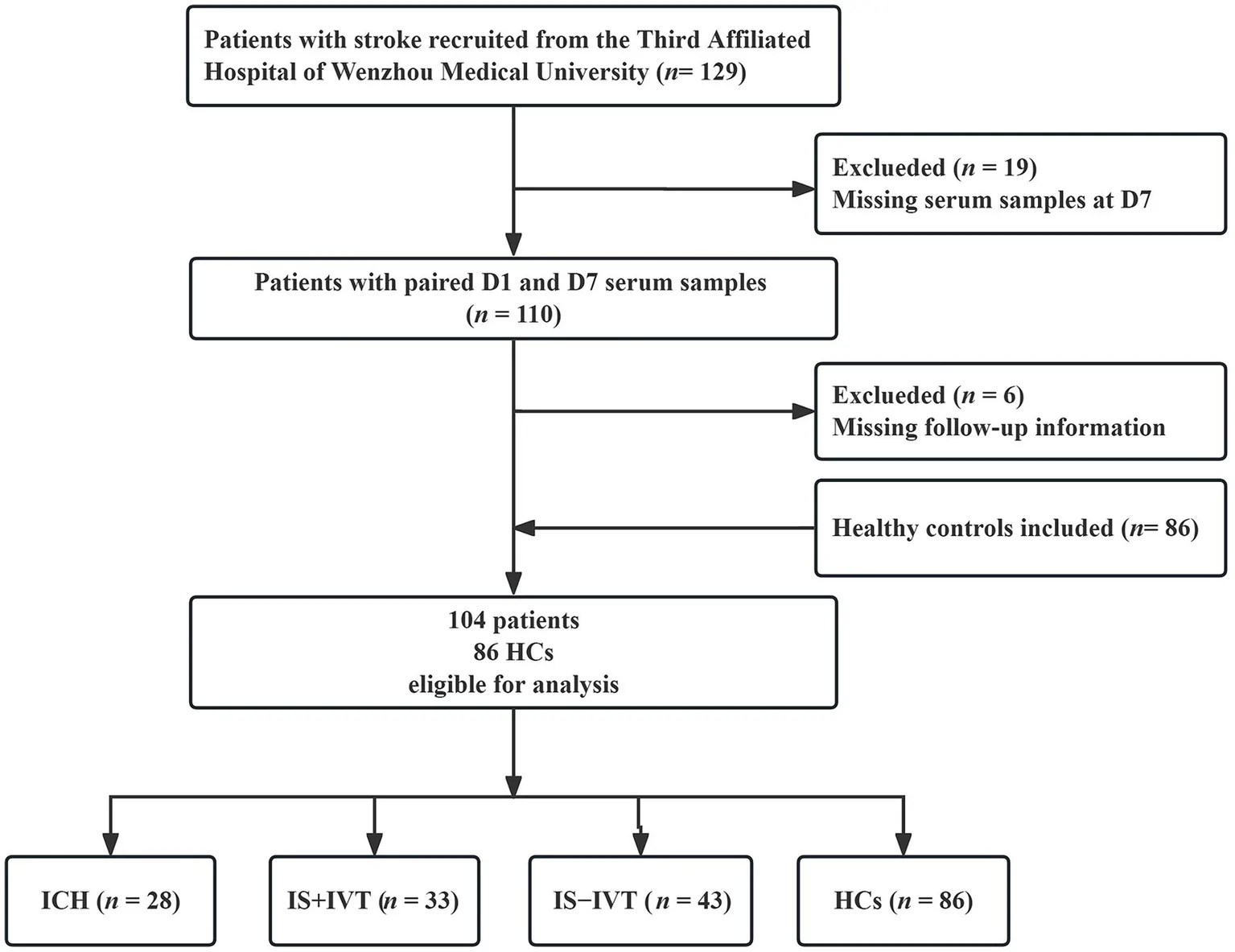
Flow diagram of participant screening, enrollment, and analytic grouping. D1, day 1 within 24 h after symptom onset; D7, day 7 after onset; HCs, healthy controls; ICH, intracerebral hemorrhage; IS+IVT, ischemic stroke with intravenous thrombolysis; IS−IVT, ischemic stroke without intravenous thrombolysis.

### 2.4 Blood sample collection and sNfL measurement

For patients with stroke, 3–5 mL of peripheral venous blood was collected at D1 and D7. D1 was defined as within 24 h after stroke onset. To minimize additional venipuncture and patient discomfort, D1 sampling was coordinated with routine clinical blood collection. When the next-morning blood draw (04:00–06:30) fell within 24 h after onset, samples were collected at that time; otherwise, blood was collected during the same-day afternoon collection period (16:00–18:00) or immediately, as needed to remain within the 24 h window. In the IS+IVT group, all D1 samples were collected within 24 h after onset and after IVT. For HCs, peripheral venous blood was collected once. Blood samples were collected in vacuum tubes without anticoagulant, allowed to stand at room temperature for approximately 30 min, and then centrifuged at 3000 r/min for 10 min to separate serum. Serum samples were aliquoted and stored at −80 °C until batch analysis. Samples underwent no more than one freeze–thaw cycle before measurement. sNfL concentrations were measured using a double-antibody sandwich chemiluminescent immunoassay on a Shine i2910 fully automated analyzer, with an NfL assay kit from Nanjing Vazyme Medical Technology Co., Ltd. (Nanjing, China; lot 250327A). The analyzer was calibrated before use according to the manufacturer’s instructions. Calibrators and quality-control materials were included in the assay procedure, and sNfL concentrations were calculated from the calibration curve. All samples were analyzed using a standardized procedure, with reagents stored as recommended and used within their expiry dates. Laboratory personnel performing the sNfL measurements were blinded to clinical stroke group and functional outcomes.

### 2.5 Baseline data collection

Demographic and clinical data were collected for all participants, including age, sex, height, weight, and laboratory test results. For patients with stroke, medical history and vascular risk factors were also recorded, including hypertension, diabetes mellitus, hyperlipidemia, smoking history, alcohol consumption history, and previous stroke history. Previous stroke history referred to stroke occurring more than 6 months before the index stroke. Cranial imaging data were reviewed. Neurologists assessed stroke severity at admission using the NIHSS (25). For patients with IS, they also classified stroke etiology according to the Trial of Org 10172 in Acute Stroke Treatment (TOAST) classification (26) and assessed the presence of acute large-vessel occlusion (LVO).

### 2.6 Functional outcome assessment

Trained research personnel assessed functional outcome by telephone at 3 and 6 months after stroke onset. Neurological recovery was recorded using the mRS, a global functional outcome scale widely used in stroke research that reflects disability and independence in activities of daily living. Scores range from 0 to 6, with higher scores indicating more severe functional impairment (27). A poor functional outcome was defined as an mRS score >2.

### 2.7 Statistical analysis

All statistical analyses were performed using R software (version 4.5.2; R Core Team), mainly with the lme4, lmerTest, and rms packages. The Shapiro–Wilk test was used to assess the distribution of continuous variables. Continuous variables are presented as medians with interquartile ranges (IQRs), and categorical variables as frequencies and percentages. Continuous variables were compared between groups using the Mann–Whitney U test or Kruskal–Wallis test, as appropriate. Categorical variables were compared using Pearson’s *χ*^2^ test or Fisher’s exact test. Bonferroni correction was applied separately to the three pairwise comparisons for each variable. Paired D1 and D7 sNfL levels were compared using the Wilcoxon signed-rank test. Except for interaction analyses, all tests were two-sided, and *p* < 0.05 was considered statistically significant, using adjusted *p*-values where applicable.

After natural log transformation of sNfL, linear mixed-effects models were used to evaluate longitudinal trajectories across the clinical stroke groups. Models were adjusted for age and admission NIHSS score and included a random intercept for participant to account for within-participant correlation; sensitivity analyses additionally adjusted for sex.

The primary endpoint was poor functional outcome at 3 months after stroke, and the secondary endpoint was poor functional outcome at 6 months. Multivariable logistic regression models were constructed to assess the associations between sNfL levels and functional outcome. Given the limited number of poor outcome events, covariate adjustment was restricted to age and admission NIHSS score. These covariates were prioritized because of their established prognostic relevance in IS and ICH (28, 29). Age is also associated with sNfL concentrations (30), while admission NIHSS quantifies initial neurological deficit severity and has been associated with sNfL levels in IS (22). sNfL was entered into the models per 10-pg/mL increase.

Restricted cubic spline (RCS) and sNfL × clinical stroke group interaction analyses were considered exploratory and hypothesis-generating. Within the multivariable logistic regression framework, RCS models with three knots located at the 10th, 50th, and 90th percentiles were used to explore the shape of the sNfL-outcome associations. Overall associations were assessed using likelihood ratio tests, and nonlinearity using Wald tests. Effect modification was assessed by including an sNfL × clinical stroke group interaction term in the multivariable logistic regression models. Four prespecified interaction tests were performed, and after Bonferroni correction, the threshold for statistical significance was set at *α* = 0.0125. Exploratory pairwise comparisons of group-specific associations were Tukey-adjusted and were interpreted as secondary to the Bonferroni-corrected overall interaction tests.

## 3 Results

### 3.1 Baseline characteristics of the study participants

The study included 104 patients with acute stroke and 86 healthy controls (HCs). The stroke and HC groups did not differ significantly in age (68.0 years vs. 63.0 years, *p* = 0.105) or sex distribution (men, 69.2% vs. 57.0%; *p* = 0.110), indicating that the two groups were broadly comparable in demographic characteristics (Supplementary Table S1). Compared with HCs, patients with stroke had significantly higher D1 sNfL levels [56.8 (IQR, 31.9–99.8) pg/mL vs. 24.4 (IQR, 15.8–40.2) pg/mL; *p* < 0.001], indicating elevated peripheral sNfL within 24 h after acute stroke.

No significant differences were observed among the three clinical stroke groups in age, sex, hypertension, hyperlipidemia, smoking history, alcohol consumption history, previous stroke history, D1 sNfL levels, or onset-to-D1 sampling intervals (all *p* > 0.05; Table 1). The prevalence of diabetes mellitus differed significantly among the groups; further pairwise comparisons showed that only the difference between the IS+IVT and ICH groups remained significant after Bonferroni correction (57.6% vs. 21.4%; Bonferroni-corrected *p* = 0.025). Admission NIHSS scores differed significantly among the three groups overall (*p* = 0.034), but no pairwise comparison reached statistical significance after Bonferroni correction (Bonferroni-corrected *p* > 0.05). For onset-to-D1 sampling intervals, all three pairwise comparisons were nonsignificant after Bonferroni correction (Bonferroni-corrected *p* = 1.000). Among the 76 patients with IS, TOAST classification identified large-artery atherosclerosis in 42 (55.3%), small-vessel occlusion in 13 (17.1%), cardioembolism in 11 (14.5%), and an undetermined etiology in 10 (13.2%). Acute LVO was present in 14 patients (18.4%), including 9 in the IS+IVT group and 5 in the IS−IVT group (Supplementary Table S6).

**Table 1:** Baseline characteristics of patients with acute stroke by clinical stroke group.

Characteristics	ICH (*n* = 28)	IS+IVT (*n* = 33)	IS−IVT (*n* = 43)	*p*-value
Demographics				
Age (years)	65.0 (52.0–70.0)	69.0 (59.0–77.0)	68.0 (54.0–72.5)	0.238
Sex (male), *n* (%)	20 (71.4)	19 (57.6)	33 (76.7)	0.191
Medical history				
Hypertension, *n* (%)	27 (96.4)	29 (87.9)	40 (93.0)	0.531
Diabetes, *n* (%)	6 (21.4)	19 (57.6)	18 (41.9)	0.017
Hyperlipidemia, *n* (%)	8 (28.6)	15 (45.5)	23 (53.5)	0.117
Smoking history, *n* (%)	13 (46.4)	10 (30.3)	18 (41.9)	0.400
Alcohol consumption, *n* (%)	12 (42.9)	11 (33.3)	17 (39.5)	0.735
Prior stroke, *n* (%)	2 (7.1)	9 (27.3)	5 (11.6)	0.076
Disease severity				
Admission NIHSS	6.0 (2.8–11.3)	4.0 (2.0–6.0)	3.0 (2.0–5.5)	0.034
Sampling timing				
Onset-to-D1 sampling interval (h)	20.9 (17.9–23.2)	20.5 (18.3–22.5)	21.0 (18.0–23.0)	0.904
Biomarker				
D1 sNfL (pg/mL)	67.4 (41.1–99.2)	40.6 (24.2–92.4)	60.5 (36.2–107.3)	0.211

Data are presented as median (interquartile range) or n (%). *p*-values were calculated using the Kruskal–Wallis test for continuous variables and Pearson’s chi-square test or Fisher’s exact test for categorical variables, as appropriate. Pairwise comparisons, when performed, were adjusted using the Bonferroni method for the three comparisons within each variable and are reported in the text. D1 was defined as within 24 h after symptom onset. All D1 samples in the IS+IVT group were collected after IVT.

D1, day 1 within 24 h after symptom onset; ICH, intracerebral hemorrhage; IS+IVT, ischemic stroke with intravenous thrombolysis; IS−IVT, ischemic stroke without intravenous thrombolysis; NIHSS, National Institutes of Health Stroke Scale; sNfL, serum neurofilament light chain.

### 3.2 Dynamic changes in sNfL after stroke

During the first week after acute stroke, both D1 and D7 sNfL levels were significantly higher than those in HCs (both *p* < 0.001; Figure 2). Paired comparison showed a significant increase in sNfL from D1 to D7 (*p* < 0.001), with a large effect size (*r* = 0.82; Figure 3). Quantitatively, the median sNfL concentration increased from 56.8 pg/mL (IQR, 31.9–99.8) on D1 to 208.4 pg/mL (IQR, 104.9–375.4) on D7, corresponding to an approximately 3.7-fold increase (Figure 3). The IQR at D7 was wider than that at D1, indicating greater interindividual variability in the evolution of neuroaxonal injury during the acute phase.

**Figure 2:**
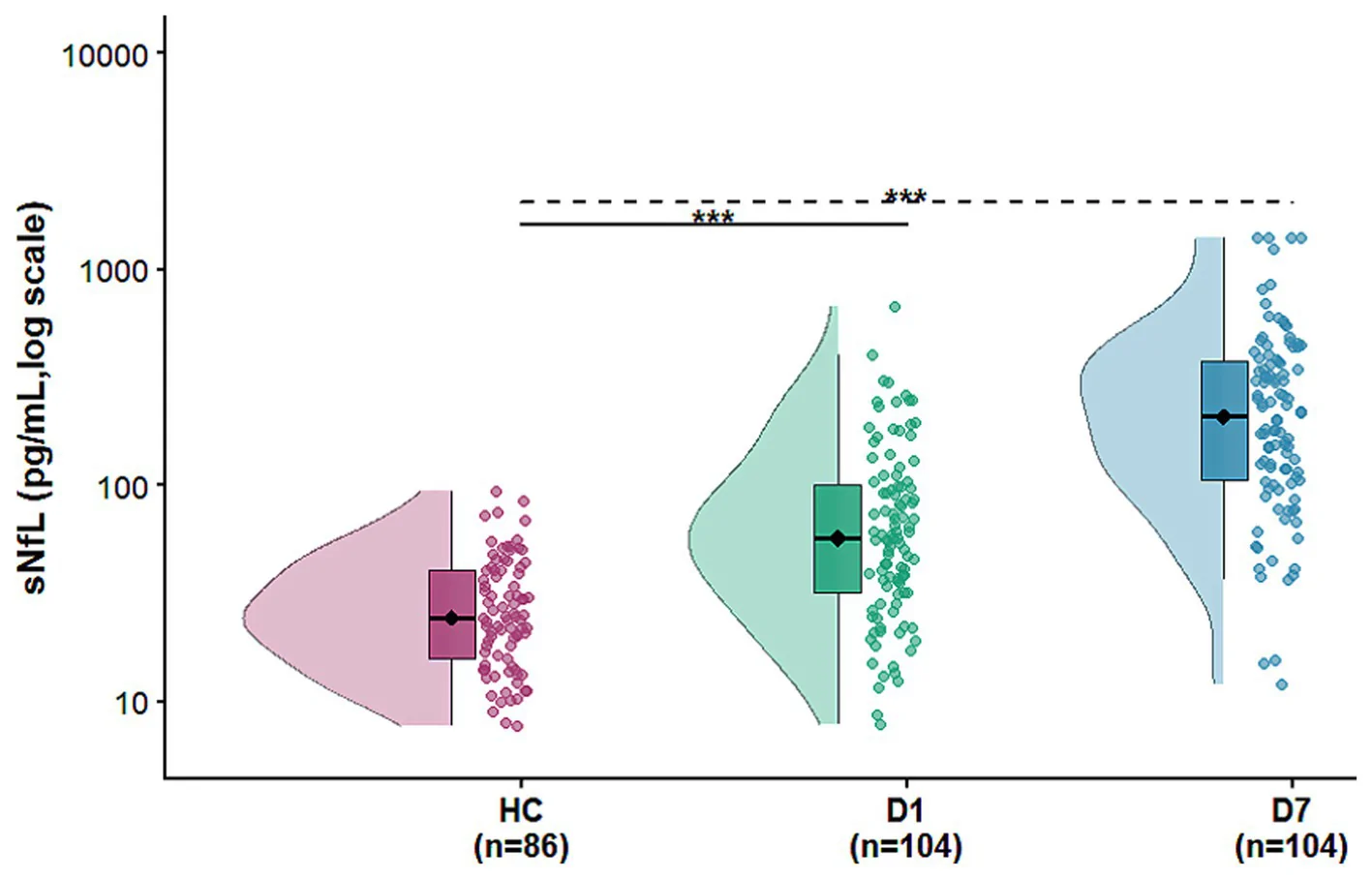
Distribution of serum neurofilament light chain levels in healthy controls and patients with acute stroke at D1 and D7. Violin plots, boxplots, and individual data points show sNfL concentrations in healthy controls and in patients with acute stroke at D1 and D7. The y-axis is displayed on a logarithmic scale. Asterisks indicate significant differences compared with healthy controls. ***p < 0.001. D1, day 1 within 24 h after symptom onset; D7, day 7 after onset; HC, healthy control; sNfL, serum neurofilament light chain.

**Figure 3:**
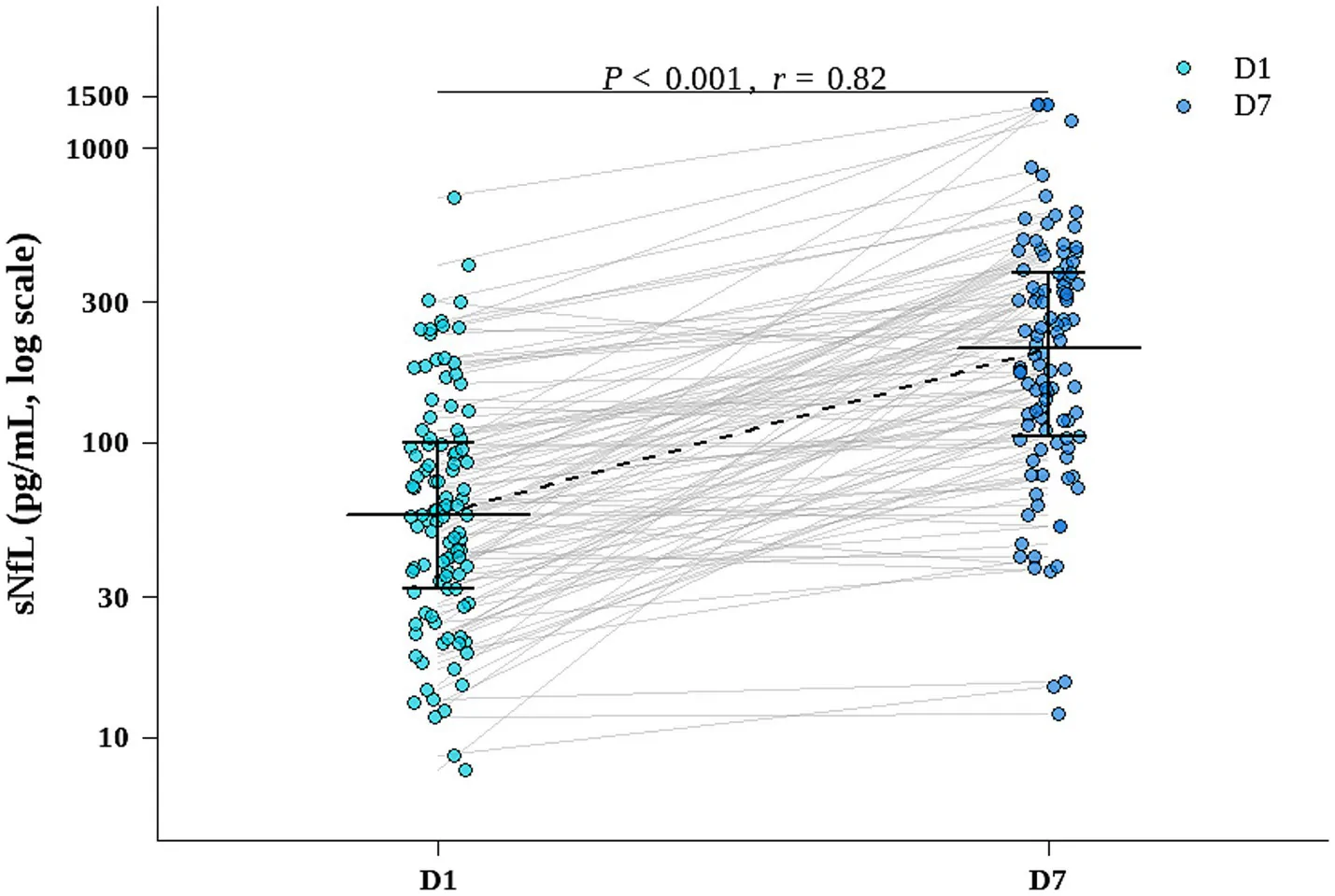
Paired changes in serum neurofilament light chain levels from D1 to D7 after acute stroke. Individual paired sNfL measurements are shown for 104 patients with acute stroke. Gray lines connect paired measurements from the same patient. Horizontal black bars indicate median values. The dashed line connects median values at D1 and D7. The y-axis is displayed on a logarithmic scale. The *p* value was calculated using the Wilcoxon signed-rank test, and r denotes the effect size. D1, day 1 within 24 h after symptom onset; D7, day 7 after onset; sNfL, serum neurofilament light chain.

Further stratified analyses showed that D7 sNfL levels were significantly higher than D1 levels in the ICH, IS+IVT, and IS−IVT groups (all *p* < 0.001; Supplementary Table S2), suggesting that the D1-to-D7 increase was observed across all three clinical stroke groups. In analyses not adjusted for confounders, D7 sNfL levels and ΔsNfL (D7 − D1) differed significantly among the three groups (*p* = 0.011 and *p* = 0.015, respectively), with the most pronounced increase observed in the ICH group. However, in the repeated-measures linear mixed-effects model adjusted for age and admission NIHSS score, the main effect of time was highly significant [*F*(1, 101) = 162.50, *p* < 0.001], whereas neither the main effect of group (*p* = 0.228) nor the time × group interaction (*p* = 0.225) was statistically significant (Figure 4; Table 2; Supplementary Table S3). Covariate analysis further showed that admission NIHSS score was independently and positively associated with ln(sNfL) levels (*β* = 0.058, *p* = 0.002; Table 2). These adjusted results suggest that the unadjusted between-group differences may have reflected imbalance in admission neurological deficit severity rather than an independent effect of clinical stroke group on sNfL trajectories. Sensitivity analysis with additional adjustment for sex yielded results consistent with the main analysis (Supplementary Table S3).

**Figure 4:**
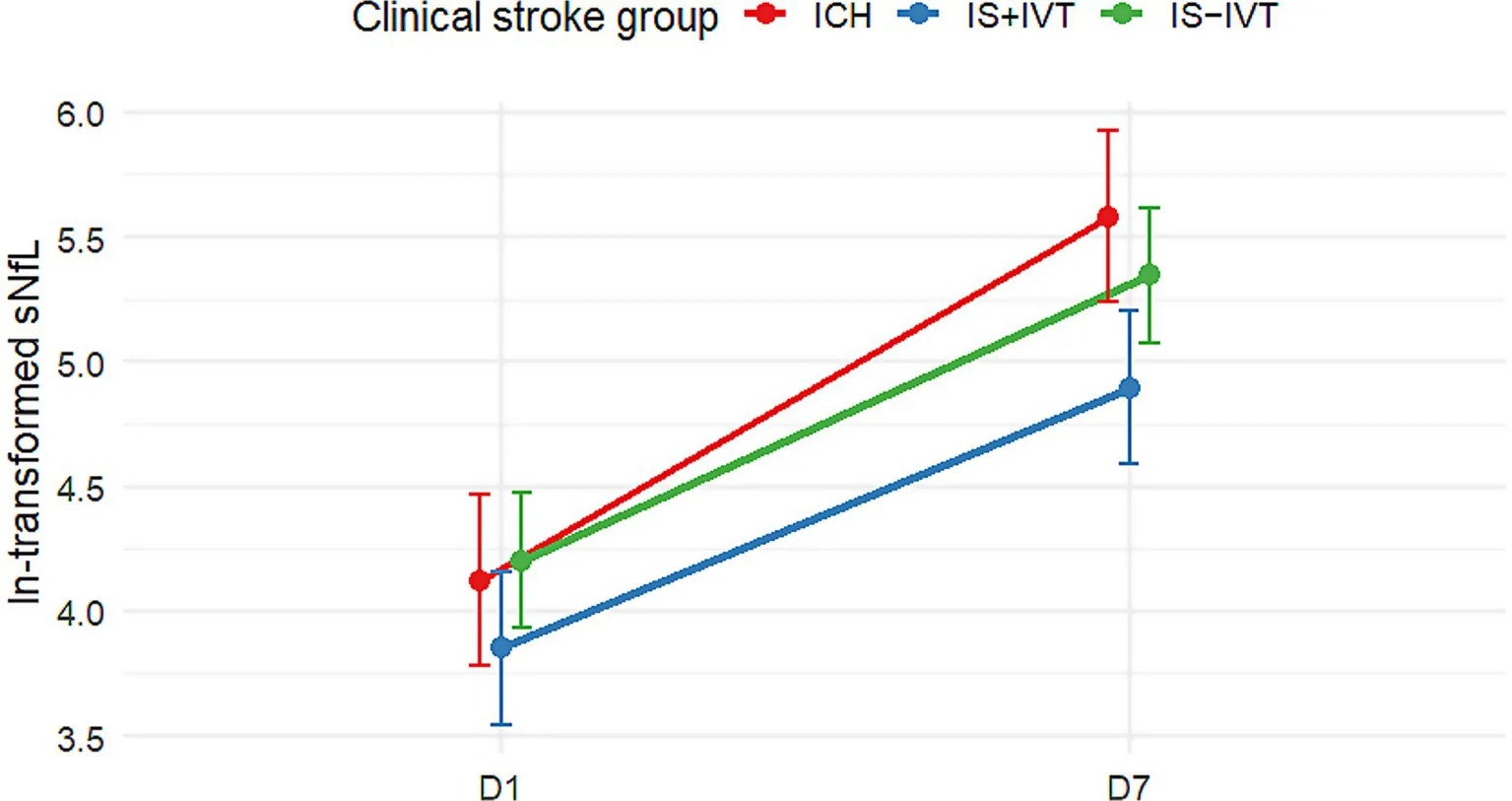
Adjusted longitudinal trajectories of ln-transformed serum neurofilament light chain by clinical stroke group. Points and error bars represent model-based estimated marginal means and 95% confidence intervals of ln-transformed sNfL at D1 and D7 by clinical stroke group. Estimates were derived from a linear mixed-effects model adjusted for age and admission NIHSS score, with a random intercept for participant. D1, day 1 within 24 h after symptom onset; D7, day 7 after onset; ICH, intracerebral hemorrhage; IS+IVT, ischemic stroke with intravenous thrombolysis; IS−IVT, ischemic stroke without intravenous thrombolysis; NIHSS, National Institutes of Health Stroke Scale; sNfL, serum neurofilament light chain.

**Table 2:** Adjusted linear mixed-effects model for longitudinal changes in ln-transformed serum neurofilament light chain after acute stroke.

Variable	*β*	SE	*t*-value	*p*-value
Control variables				
Age	0.011	0.006	1.76	0.081
Admission NIHSS	0.058	0.019	3.12	0.002
Main effects				
Time (D7 vs. D1)	1.455	0.181	8.05	<0.001
Group (IS+IVT vs. ICH)	−0.271	0.235	−1.16	0.250
Group (IS−IVT vs. ICH)	0.077	0.225	0.34	0.733
Interaction effects				
Time × Group (IS+IVT)	−0.412	0.246	−1.68	0.096
Time × Group (IS−IVT)	−0.311	0.232	−1.34	0.183
Intercept	3.083	0.471	6.55	<0.001

The dependent variable was natural log-transformed sNfL, with estimates reported on the ln(sNfL) scale. The model included fixed effects for time, clinical stroke group, time × group interaction, age, and admission NIHSS score, with a random intercept for participant. ICH and D1 served as the reference categories. The interaction terms represent differences in D1-to-D7 change compared with the ICH group.

*β*, regression coefficient; SE, standard error; D1, day 1 within 24 h after symptom onset; D7, day 7 after onset; ICH, intracerebral hemorrhage; IS+IVT, ischemic stroke with intravenous thrombolysis; IS−IVT, ischemic stroke without intravenous thrombolysis; NIHSS, National Institutes of Health Stroke Scale; sNfL, serum neurofilament light chain.

### 3.3 Association between sNfL and poor functional outcome

Poor functional outcome (mRS > 2) occurred in 22 of 104 patients (21.2%) at 3 months and 16 of 104 (15.4%) at 6 months. The full mRS distributions are provided in Supplementary Table S7. Multivariable logistic regression models were constructed to further assess the association between sNfL levels and functional outcome. After adjustment for age and admission NIHSS score, D1 sNfL remained independently associated with poor functional outcome at both 3 months (OR = 1.05; 95% CI, 1.00–1.12; *p* = 0.047) and 6 months (OR = 1.07; 95% CI, 1.01–1.14; *p* = 0.025). Each 10-pg/mL increase in D1 sNfL corresponded to approximately 5% and 7% higher odds of poor functional outcome, respectively (Table 3). By contrast, D7 sNfL was not significantly associated with poor functional outcome at either 3 months or 6 months in the prespecified linear logistic models adjusted for age and admission NIHSS score (both *p* > 0.05; Table 3). These results suggest that D1 sNfL, but not D7 sNfL, may provide early prognostic information for functional outcome at short- and medium-term follow-up after stroke.

**Table 3:** Multivariable associations between serum neurofilament light chain levels and poor functional outcome at 3 and 6 months after stroke.

Biomarker	Follow-up time	Adjusted OR (95% CI)	*p*-value
D1 sNfL	3 months	1.05 (1.00–1.12)	0.047
	6 months	1.07 (1.01–1.14)	0.025
D7 sNfL	3 months	1.01 (0.99–1.03)	0.191
	6 months	1.01 (1.00–1.03)	0.088

Odds ratios are expressed per 10 pg/mL increase in sNfL. Models were adjusted for age and admission NIHSS score. Poor functional outcome was defined as a modified Rankin Scale score >2 at the corresponding follow-up time. Estimates, confidence intervals, and p values were calculated using unrounded values.

CI, confidence interval; D1, day 1 within 24 h after symptom onset; D7, day 7 after onset; mRS, modified Rankin Scale; NIHSS, National Institutes of Health Stroke Scale; OR, odds ratio; sNfL, serum neurofilament light chain.

### 3.4 Dose–response relationship between sNfL and poor functional outcome

Exploratory RCS analyses were used to describe the relationships of D1 and D7 sNfL with poor functional outcome (Figure 5). D1 sNfL showed an overall association with poor functional outcome at both 3 months and 6 months (*p* = 0.044 and *p* = 0.027, respectively), whereas tests for nonlinearity were not statistically significant (*p* = 0.216 and *p* = 0.350, respectively). Although the fitted D1 curves appeared approximately linear, the nonsignificant tests for nonlinearity did not establish linearity. In contrast, D7 sNfL showed an exploratory nonlinear pattern in relation to poor functional outcome at 3 months (*P* for nonlinearity = 0.034). Derivative-based exploratory assessment of the fitted spline curve described a potential turning point at approximately 477.4 pg/mL, after which the estimated curve tended to plateau in the high-value range. No significant nonlinearity was observed between D7 sNfL and 6-month poor functional outcome (*P* for nonlinearity = 0.101). The overall RCS association for D7 sNfL at 6 months was borderline, whereas the prespecified linear logistic model and the nonlinearity test were not statistically significant. Given the limited number of poor outcome events, all RCS findings should be regarded as exploratory; the potential turning point was descriptive and requires validation in larger cohorts.

**Figure 5:**
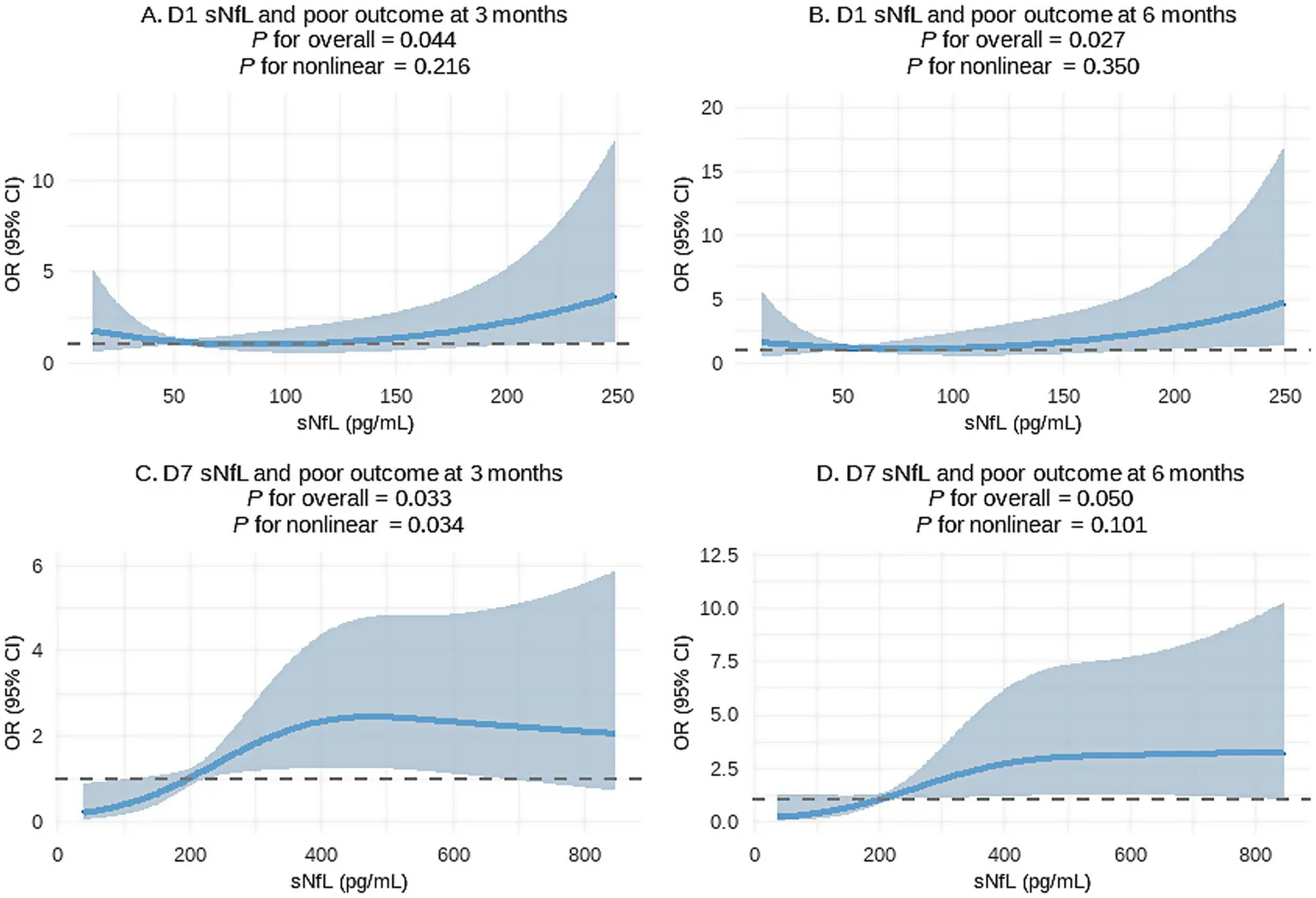
Restricted cubic spline analyses of the associations between serum neurofilament light chain levels and poor functional outcome at 3 and 6 months after stroke. Restricted cubic spline curves show adjusted odds ratios and 95% confidence intervals for poor functional outcome according to D1 and D7 sNfL levels. Poor functional outcome was defined as a modified Rankin Scale score >2 at the corresponding follow-up time. Models were adjusted for age and admission NIHSS score. Restricted cubic spline models used three knots placed at the 10th, 50th, and 90th percentiles of sNfL. Odds ratios were calculated relative to the median sNfL value at each time point. For visualization, spline curves are displayed over the 5th to 95th percentile range of observed sNfL values; all available observations were included in model fitting. The dashed horizontal line indicates OR = 1. Panels **(A,B)** show associations between D1 sNfL and poor functional outcome at 3 and 6 months, respectively; panels **(C,D)** show associations between D7 sNfL and poor functional outcome at 3 and 6 months, respectively. CI, confidence interval; D1, day 1 within 24 h after symptom onset; D7, day 7 after onset; mRS, modified Rankin Scale; NIHSS, National Institutes of Health Stroke Scale; OR, odds ratio; RCS, restricted cubic spline; sNfL, serum neurofilament light chain.

### 3.5 Effect modification by clinical stroke group

In the interaction analyses, after Bonferroni correction (*α* = 0.0125), the *p*-values for all four interaction terms between sNfL and clinical stroke group exceeded the corrected significance threshold (Supplementary Table S4). Pairwise comparisons using Tukey’s method also showed no significant differences in effect estimates among the three clinical stroke groups (Supplementary Table S5). These results indicate that there was no statistical evidence of effect modification by clinical stroke group in the association between sNfL and poor functional outcome.

## 4 Discussion

In this prospective cohort study, we evaluated the early dynamics of sNfL during the first week after acute stroke and examined its prognostic relevance. Our main findings are twofold. First, sNfL levels increased significantly during the first week after stroke, and D1 sNfL levels were already higher in patients with stroke than in HCs. Second, after adjustment for age and admission NIHSS score, D1 sNfL remained independently associated with poor functional outcome at 3 and 6 months, whereas the association for D7 sNfL was attenuated. These findings suggest that the prognostic interpretation of sNfL is time-dependent, and that sampling time may be an important consideration for its clinical application.

We first observed a sustained increase in sNfL during the first week after acute stroke, with D1 levels already elevated compared with those in HCs. This pattern is consistent with previous longitudinal studies reporting that NfL continues to increase from several days to weeks after stroke onset (19–21). From a pathophysiological perspective, this increase may reflect not only the delayed release of NfL after initial axonal injury, but also ongoing secondary injury processes during the early poststroke period, including oxidative stress, amplified inflammatory responses, and blood–brain barrier dysfunction (31). Because these secondary injury processes may vary substantially among individuals, D7 sNfL levels may integrate more heterogeneous pathological information than D1 levels. This may partly explain why the prognostic association of D7 sNfL was less stable than that of D1 sNfL.

We found no independent difference in longitudinal sNfL trajectories among the three clinical stroke groups. The predominantly mild-to-moderate severity profile and limited representation of severe stroke may have reduced our ability to detect differences in these trajectories. Patients in the ICH, IS+IVT, and IS−IVT groups all showed a broadly similar increase during the first week after onset. During this early period, sNfL dynamics may be driven more by shared time-dependent injury processes than by specific clinical group membership. Regardless of whether the primary event is ischemia or hemorrhage, acute stroke can induce axonal injury and sustained NfL release (9, 32); secondary mechanisms such as oxidative stress, inflammation, and blood–brain barrier dysfunction may also persist for several days after onset and contribute to rising sNfL levels (8, 31, 32). Because sNfL more closely reflects the overall burden of axonal injury than the specific mode of injury, the time effect remained significant after adjustment for age and admission NIHSS score, whereas the group effect and time × group interaction did not reach statistical significance. Descriptively, the D1-to-D7 increase in sNfL appeared smaller in the IS+IVT group than in the IS−IVT group. However, the adjusted between-group difference was not statistically significant, and D1 samples in the IS+IVT group were obtained after thrombolysis. Given the observational design and limited sample size, this finding should be interpreted as exploratory and should not be attributed to a treatment effect of IVT. Further investigation in larger cohorts with pretreatment sampling is needed.

The present study suggests that D1 sNfL may be an early prognostic biomarker for short- and medium-term functional outcome after stroke. In models adjusted for age and admission NIHSS score, higher D1 sNfL levels remained associated with increased odds of poor functional outcome at both 3 and 6 months. Mechanistically, D1 sNfL may reflect early neuroaxonal injury during the hyperacute-to-acute phase of stroke, while later measurements may increasingly reflect delayed NfL release and secondary injury processes (7, 9, 22). In contrast, the NIHSS score mainly captures the clinical severity of neurological deficits and can be influenced by lesion location and the scale’s differential sensitivity to specific neurological domains (33, 34). Although NIHSS scoring is generally reliable when performed by trained raters, score differences between provider groups can still occur in real-world clinical practice (35). Therefore, D1 sNfL may provide prognostic information not fully captured by NIHSS score. This finding is consistent with Uphaus et al., who reported that acute-phase sNfL independently predicted unfavorable functional outcome after IS in multivariable analysis (22). Zheng et al. similarly showed that early plasma NfL was independently associated with long-term mortality and neurological outcomes in patients with ICH (21). By reproducing these associations in a heterogeneous acute stroke cohort including ICH and IS patients with and without IVT, our findings suggest that the prognostic relevance of early sNfL can be examined across these clinical stroke groups, although our interaction analyses did not provide definitive evidence of group-specific differences.

Functional outcome after ischemic stroke is associated with infarct volume and lesion location, including involvement of motor pathways (36). In a small exploratory study during post-stroke rehabilitation, Lanzone et al. (37) reported an association between plasma NfL and lesion volume, while EEG spectral exponent measures were related to clinical scores and differed according to cortical involvement. These observations suggest that molecular and electrophysiological measures may capture complementary aspects of stroke injury and recovery, highlighting the importance of anatomical and functional context when interpreting sNfL-outcome associations.

By contrast, D7 sNfL was not significantly associated with poor functional outcome after multivariable adjustment. This finding should first be interpreted in light of limited statistical power: only 22 patients had poor functional outcome at 3 months, and the ability to detect modest independent associations was reduced once multiple covariates were included. In addition, biological heterogeneity may have contributed to the attenuation of the association. D7 sNfL levels may reflect more heterogeneous post-acute injury processes, including delayed neuroaxonal release, secondary neuroinflammation, and blood–brain barrier dysfunction, rather than the initial burden of axonal injury alone (31, 32). These processes may vary across individuals and weaken the stability of the association between D7 sNfL and later functional outcome. Moreover, admission NIHSS score already captures a substantial component of stroke severity; after adjustment for NIHSS score, the additional independent prognostic information provided by D7 sNfL may be limited.

The exploratory RCS analyses provided a complementary view of the sNfL-outcome associations. D1 sNfL showed an overall association with poor functional outcome at both 3 and 6 months, and tests for nonlinearity were not statistically significant. Although the fitted D1 curves appeared approximately linear, the limited number of outcome events reduces the ability to detect departures from linearity. In contrast, D7 sNfL showed an exploratory nonlinear pattern only for 3-month poor functional outcome, with the estimated risk increase tending to plateau at higher sNfL concentrations. This finding suggests that the prognostic association of D7 sNfL may be less stable than that of D1 sNfL and more susceptible to variation at high concentrations. This nonlinear pattern is consistent with the possibility that D7 sNfL may reflect secondary neurological injury rather than a linear accumulation of primary axonal damage. These processes may not contribute equivalently to long-term functional outcome, resulting in a flattened risk curve at higher D7 sNfL levels. Given the limited number of poor outcome events, all RCS findings should be regarded as exploratory, and the proposed biological explanation for the D7 pattern remains speculative. The estimated turning point should not be directly extrapolated as a clinical threshold and requires validation in larger independent cohorts.

The exploratory interaction analyses did not detect significant effect modification by clinical stroke group. After Bonferroni correction, the *p* values for all four interaction terms were well above the corrected threshold, ranging from 0.313 to 0.946. Zheng et al. reported an independent association between sNfL and outcome in ICH, and Uphaus et al. reported a similar association in IS (21, 22). Although the direction of these findings was consistent, direct comparison across clinically relevant stroke groups had not previously been performed within the same cohort. The design of the present study allowed such a comparison under a common analytical framework, and we found no statistical evidence that the association between sNfL and poor outcome differed by clinical stroke group. However, interaction tests require substantially larger sample sizes than main-effect analyses. Given the size of the present cohort, the analyses were underpowered to reliably exclude small or moderate group-specific differences. Therefore, the negative interaction findings should be interpreted as an absence of evidence for significant effect modification, rather than definitive evidence that such modification does not exist.

This study has several methodological strengths. First, the study used a prospective design with predefined inclusion and exclusion criteria. Second, the study included three clinically relevant stroke groups—ICH, IS+IVT, and IS−IVT—addressing the limitation that many previous sNfL studies focused on a single stroke type and enabling cross-group comparison under a common analytical framework. Third, paired longitudinal sampling at D1 and D7 allowed direct comparison of prognostic information from two time points within the same cohort, rather than relying on indirect inference across studies. In addition, the combined use of repeated-measures linear mixed-effects models, RCS analyses, and effect modification analyses allowed sNfL to be assessed from multiple perspectives, including longitudinal trajectories, dose–response relationships, and group differences.

Several limitations should also be considered. First, this small, single-center cohort was recruited from a neurology ward within 24 h after onset and predominantly comprised patients with mild-to-moderate stroke. Hospital triage and admission practices for patients requiring thrombectomy, neurosurgical management, or intensive care, together with delays in neurology ward admission, contributed to the underrepresentation of severe stroke and limit the generalizability of our findings. Exclusion of 19 patients without D7 samples may also have introduced selection bias into the D1 prognostic analyses, because sample availability may be associated with clinical severity and recovery. The direction and magnitude of this bias remain uncertain. Second, sNfL was measured only at D1 and D7, which precluded characterization of earlier kinetic changes and the complete peak trajectory. Third, the prognostic models were adjusted only for age and admission NIHSS score. Lesion burden, lesion topography, and treatment-related factors therefore remain potential sources of residual confounding. With only 22 and 16 poor outcome events at 3 and 6 months, respectively, the prognostic models remain susceptible to overfitting and imprecise estimates despite restricted covariate adjustment. Dichotomizing the mRS may also obscure clinically meaningful differences in domain-specific recovery, particularly in patients with mild stroke. The prognostic findings should therefore be interpreted as preliminary associations in patients who completed paired sampling and follow-up.

## 5 Conclusion

In this prospective cohort, sNfL levels increased during the first week after acute stroke, and levels within 24 h after onset were already higher than those in HCs. Higher D1 sNfL was associated with poor functional outcome at 3 and 6 months after adjustment for age and admission NIHSS score, whereas the associations for D7 sNfL were attenuated after adjustment. Adjusted longitudinal analyses did not detect significant differences in sNfL trajectories among the three clinical stroke groups. Overall, these findings suggest potential prognostic relevance of early sNfL measurement in acute stroke, although their generalizability is limited by the restriction to patients with paired sampling and follow-up. Further evaluation in larger, multicenter cohorts with standardized sampling protocols is needed before clinical application.

## Data Availability

The raw data supporting the conclusions of this article will be made available by the authors, without undue reservation.
