# Supplementary material for: Longitudinal dynamics of serum neurofilament light chain and prognostic value for functional outcome after acute stroke: a prospective cohort study
Source: Front Neurol. 2026 Oct 5;17:1932102. doi: 10.3389/fneur.2026.1932102 (PMC13677471; doi:10.3389/fneur.2026.1932102)
Supplement: Supplementary file 1 [file Supplementary_file_1.docx]

**SUPPLEMENTARY MATERIALS**

**Supplementary Table 1.** Baseline characteristics of patients with acute stroke and healthy controls

| **Characteristic** | **Stroke group**  **(*n* = 104)** | **HCs**  **(*n* = 86)** | ***P value*** |
| --- | --- | --- | --- |
| **Demographics** |  |  |  |
| Age (years) | 68.0 (54.8–73.2) | 63.0 (51.2–72.0) | 0.105 |
| Sex (male), *n* (%) | 72 (69.2%) | 49 (57.0%) | 0.110 |
| **Biomarker** |  |  |  |
| sNfL(pg/mL) | 56.8 (31.9–99.8) | 24.4 (15.8–40.2) | <0.001 |

**Note:** Data are presented as median (interquartile range) or n (%). For patients with stroke, sNfL values refer to D1 measurements obtained within 24 h after symptom onset. P values were calculated using the Mann–Whitney U test for continuous variables and Pearson's chi-square test or Fisher's exact test for categorical variables, as appropriate.

**Abbreviations:** D1, day 1 within 24 h after symptom onset; HCs, healthy controls; sNfL, serum neurofilament light chain.

**Supplementary Table 2.** Dynamic changes in sNfL from D1 to D7 by clinical stroke group

| **Group** | ***n*** | **D1 sNfL (pg/mL)** | **D7 sNfL (pg/mL)** | **ΔsNfL (pg/mL)** | **Within‑group *P* value** |
| --- | --- | --- | --- | --- | --- |
| **ICH** | 28 | 67.4 (41.1–99.2) | 331.3 (177.8–445.4) | 243.7 (136.9–383.5) | <0.001 |
| **IS+IVT** | 33 | 40.6 (24.2–92.4) | 121.7 (51.9–326.9) | 75.6 (11.1–249.3) | <0.001 |
| **IS**−**IVT** | 43 | 60.5 (36.2–107.3) | 172.5 (125.1–311.2) | 107.9 (38.2–280.0) | <0.001 |
| **Overall *P* value** |  | 0.211 | 0.011 | 0.015 |  |

**Note:** Data are presented as median (interquartile range). ΔsNfL was calculated as D7 sNfL minus D1 sNfL. Within-group *P* values were calculated using the Wilcoxon signed-rank test. Overall *P* values were calculated using the Kruskal–Wallis test.

**Abbreviations:** D1, day 1 within 24 h after symptom onset; D7, day 7 after onset; ICH, intracerebral hemorrhage; IS+IVT, ischemic stroke with intravenous thrombolysis; IS−IVT, ischemic stroke without intravenous thrombolysis; sNfL, serum neurofilament light chain.

**Supplementary Table 3.** Sensitivity analysis of linear mixed-effects models for longitudinal ln-transformed sNfL trajectories

| Model | **Time main effect *P* value** | **Group main effect *P* value** | **Time × Group interaction *P* value** |
| --- | --- | --- | --- |
| Primary analysis | <0.001 | 0.228 | 0.225 |
| Sensitivity analysis | <0.001 | 0.280 | 0.225 |

**Note:** The primary model was adjusted for age and admission NIHSS score. The sensitivity model was additionally adjusted for sex. P values are from fixed effects in the linear mixed-effects model of natural log-transformed sNfL.

**Abbreviations:** NIHSS, National Institutes of Health Stroke Scale; sNfL, serum neurofilament light chain.

**Supplementary Table 4.** Likelihood ratio tests for interactions between sNfL and clinical stroke group in relation to poor functional outcome

| **Interaction term** | ***P* for interaction** | **Bonferroni-corrected significance threshold** |
| --- | --- | --- |
| D1 sNfL × clinical stroke group, 3-month poor functional outcome | 0.946 | 0.0125 |
| D1 sNfL × clinical stroke group, 6-month poor functional outcome | 0.625 | 0.0125 |
| D7 sNfL × clinical stroke group, 3-month poor functional outcome | 0.555 | 0.0125 |
| D7 sNfL × clinical stroke group, 6-month poor functional outcome | 0.313 | 0.0125 |

**Note:** Interaction effects were assessed by likelihood ratio tests in multivariable logistic regression models adjusted for age and admission NIHSS score. Poor functional outcome was defined as a modified Rankin Scale score >2. Bonferroni correction was applied for four prespecified interaction tests, with a corrected significance threshold of α = 0.0125.

**Abbreviations:** D1, day 1 within 24 h after symptom onset; D7, day 7 after onset; NIHSS, National Institutes of Health Stroke Scale; sNfL, serum neurofilament light chain.

**Supplementary Table 5.** Tukey-adjusted pairwise comparisons of group-specific sNfL–outcome associations

| **Follow-up time** | **Biomarker** | **Comparison** | **Ratio of ORs (95% CI)** | ***P* value** |
| --- | --- | --- | --- | --- |
| 3 months | D1 sNfL | ICH vs IS+IVT | 0.99 (0.84–1.17) | 0.995 |
|  |  | ICH vs IS−IVT | 1.01 (0.90–1.14) | 0.970 |
|  |  | IS+IVT vs IS−IVT | 1.02 (0.88–1.19) | 0.954 |
|  | D7 sNfL | ICH vs IS+IVT | 1.02 (0.97–1.06) | 0.684 |
|  |  | ICH vs IS−IVT | 1.00 (0.96–1.04) | 0.997 |
|  |  | IS+IVT vs IS−IVT | 0.98 (0.95–1.02) | 0.622 |
| 6 months | D1 sNfL | ICH vs IS+IVT | 1.09 (0.85–1.40) | 0.778 |
|  |  | ICH vs IS−IVT | 0.97 (0.85–1.12) | 0.928 |
|  |  | IS+IVT vs IS−IVT | 0.89 (0.70–1.15) | 0.652 |
|  | D7 sNfL | ICH vs IS+IVT | 1.03 (0.97–1.09) | 0.679 |
|  |  | ICH vs IS−IVT | 0.99 (0.95–1.04) | 0.911 |
|  |  | IS+IVT vs IS−IVT | 0.97 (0.91–1.02) | 0.428 |

**Note:** Ratio of ORs denotes the ratio of group-specific odds ratios per 10 pg/mL increase in sNfL. Pairwise *P* values were Tukey-adjusted. Models were adjusted for age and admission NIHSS score. Overall interactions were evaluated using Bonferroni correction for four prespecified tests, with α = 0.0125.

**Abbreviations:** CI, confidence interval; D1, day 1 within 24 h after symptom onset; D7, day 7 after onset; ICH, intracerebral hemorrhage; IS+IVT, ischemic stroke with intravenous thrombolysis; IS−IVT, ischemic stroke without intravenous thrombolysis; NIHSS, National Institutes of Health Stroke Scale; OR, odds ratio; sNfL, serum neurofilament light chain.

**Supplementary Table 6**. Etiological classification and acute large-vessel occlusion status in patients with ischemic stroke

| **Characteristic** | **All IS (n=76)** | **IS+IVT (n=33)** | **IS-IVT (n=43)** |
| --- | --- | --- | --- |
| **TOAST classification** |  |  |  |
| Large-artery atherosclerosis, *n* (%) | 42 (55.3%) | 17 (51.5%) | 25 (58.1%) |
| Small-vessel occlusion, *n* (%) | 13 (17.1%) | 4 (12.1%) | 9 (20.9%) |
| Cardioembolism, *n* (%) | 11 (14.5%) | 8 (24.2%) | 3 (7.0%) |
| Other determined, *n* (%) | 0 (0.0%) | 0 (0.0%) | 0 (0.0%) |
| Undetermined, *n* (%) | 10 (13.2%) | 4 (12.1%) | 6 (14.0%) |
| **Acute large-vessel occlusion** |  |  |  |
| Present, *n* (%) | 14 (18.4%) | 9 (27.3%) | 5 (11.6%) |
| Absent, *n* (%) | 62 (81.6%) | 24 (72.7%) | 38 (88.4%) |

**Note:** Data are presented as n (%), with percentages calculated using the total number of patients in each column. TOAST classification and acute large-vessel occlusion status were available for all 76 patients with IS and are reported as separate characteristics. Percentages may not sum to 100 because of rounding.

**Abbreviations:** IS, ischemic stroke; IVT, intravenous thrombolysis; TOAST, Trial of Org 10172 in Acute Stroke Treatment.

**Supplementary Table 7.** Distribution of modified Rankin Scale scores at 3 and 6 months

| **mRS score** | **3 months (n=104)** | **6 months (n=104)** |
| --- | --- | --- |
| 0 | 27 (26.0%) | 30 (28.8%) |
| 1 | 34 (32.7%) | 38 (36.5%) |
| 2 | 21 (20.2%) | 20 (19.2%) |
| 3 | 15 (14.4%) | 11 (10.6%) |
| 4 | 3 (2.9%) | 1 (1.0%) |
| 5 | 4 (3.8%) | 4 (3.8%) |
| 6 | 0 (0.0%) | 0 (0.0%) |

**Note:** Data are presented as n (%). Poor functional outcome was defined as mRS >2 and occurred in 22 patients (21.2%) at 3 months and 16 (15.4%) at 6 months. Percentages may not sum to 100 because of rounding.

**Abbreviations:** mRS, modified Rankin Scale.
